# Expression of Autophagy and Mitophagy Markers in Breast Cancer Tissues

**DOI:** 10.3389/fonc.2021.612009

**Published:** 2021-08-18

**Authors:** Mohd Fazirul Mustafa, Suhainizam Muhamad Saliluddin, Sharida Fakurazi, Nur Maya Sabrina Tizen Laim, Suria Hayati Md Pauzi, Nik Hasimah Nik Yahya, Navarasi S. Raja Gopal, Maizaton Atmadini Abdullah, Sandra Maniam

**Affiliations:** ^1^Department of Human Anatomy, Faculty of Medicine and Health Sciences, Universiti Putra Malaysia, Selangor Darul Ehsan, Malaysia; ^2^Department of Community Health, Faculty of Medicine and Health Sciences, Universiti Putra Malaysia, Selangor, Malaysia; ^3^Department of Pathology, Universiti Kebangsaan Malaysia Medical Centre, Kuala Lumpur, Malaysia; ^4^Histopathology Unit, Hospital Kuala Lumpur, Kuala Lumpur, Malaysia; ^5^Department of Histopathology, Hospital Putrajaya, Putrajaya, Malaysia; ^6^Department of Pathology, Faculty of Medicine and Health Sciences, Universiti Putra Malaysia, Selangor Darul Ehsan, Malaysia

**Keywords:** breast cancer, autophagy, mitophagy, oxidative stress, immunohistochemistry

## Abstract

Mitochondria play important roles in regulating cell bioenergetics status and reactive oxygen species (ROS) generation. ROS-induced mitochondrial damage is among the main intracellular signal inducers of autophagy. Autophagy is a cellular catabolic process that regulates protein and organelle turnover, while a selective form of autophagy, mitophagy, specifically targets dysfunctional mitochondrial degradation. This study aims to measure the levels of autophagy, mitophagy, oxidative stress, and apoptosis in invasive breast carcinoma tissues using immunohistochemistry (IHC). Tissue microarrays of 76 patients with breast cancer were stained with six IHC markers (MnSOD, Beclin-1, LC3, BNIP3, Parkin, and cleaved caspase 3). The expression intensity was determined for each tumor tissue and the adjacent tumor-matched control tissues. Intermediate and strong staining scores of MnSOD, Beclin-1, LC-3, BNIP-3, and Parkin were significantly higher in tumor tissues compared to the adjacent matched control. The scoring intensity was further classified into tissues with negative staining and positive staining, which showed that positive scores of Beclin-1 and Parkin were significantly high in tumor tissues compared to other markers. Positive association was also noted between BNIP-3 and Beclin-1 as well as LC-3 and cleaved caspase-3 immunostaining. To our knowledge, this is one of the first studies that measure both mitophagy and autophagy in the same breast cancer tissues and the adjacent matched control. The findings from this study will be of great potential in identifying new cancer biomarkers and inspire significant interest in applying anti-autophagy therapies as a possible treatment for breast cancer.

## Introduction

Breast cancer is one of the leading cancers in females and was the third highest incident cancer in 2017 (1). It is a heterogenous disease and histological diagnosed as invasive ductal carcinoma, invasive lobular carcinoma with mixed ductal/lobular carcinomas, and other rare histologic findings (2). Breast cancer therapies include endocrine therapy, systemic chemotherapy, surgical resection, postoperative radiation, and molecular targeted therapy (2–4). The breast conservation, which is also known as organ saving approach, is the intended surgical standard for most clinical situations in breast cancer (5). Breast cancers categorized according to molecular subtypes are defined as luminal A-like (ER positive and/or PR positive and HER2 negative), luminal B-like (ER positive and/or PR positive/PR negative and HER2 positive), HER2-enriched type (ER negative, PR negative, and HER2 positive), and basal-like (ER, PR, and HER2 negative).

Molecular targets in breast cancer that dictate treatment options depend on the receptor-based classification, which include estrogen receptor α (ERα)-positive, progesterone receptor (PR)-positive, human epidermal growth factor receptor 2 (HER2)-enriched, and triple-negative breast cancer (TNBC) that do not express any of these three receptors (6). Standard therapies in breast cancer aim to eradicate the tumor from breast and regional lymph nodes and to prevent metastatic recurrence. Despite early detection and increasing understanding of breast cancer biology, 30% of breast cancer patients experience recurrence whereby the cancer cells display chemoresistant phenotype (7). Intra- and inter-tumor heterogeneity and metabolic rewiring are one of the factors contributing to the resistance of cancer cells to therapy. Mitochondria have long been recognized as the powerhouse of the cell and were shown to alter cancer metabolism, which enhances tumorigenesis and/or permits cancer cell adaptation to the tumor microenvironment.

Mitochondria represent the principal source of reactive oxygen species (ROS) required for autophagy induction. Autophagy has been proposed as a crucial cellular adaptation pathway that promotes tumor progression by facilitating the survival of cancer cells in response to metabolic stress such as oxygen and nutrition deprivation (8). Interestingly, autophagy serves dual roles during tumorigenesis. At the initial stage of cancer development, autophagy represents a protective response by limiting genome-damaging events. Upon tumor development, cancer cells utilize autophagy to survive under metabolic and therapeutic stress (9).

A major component of cellular control of mitochondria integrity is the specialized form of autophagy, which is known as mitophagy. Mitophagy is a highly specific form of autophagy that degrades dysfunctional or excessive mitochondria through the process of the autophagosome–lysosomal system (10). There are multiple mechanisms by which mitochondria are targeted for degradation. One of the key regulators of mitophagy is the PINK1/Parkin pathway, which is triggered by mitochondrial membrane depolarization. Upon depolarization, PINK1 accumulates at the outer mitochondrial membrane (11). PINK1 in turn recruits the E3 ubiquitin ligase, Parkin, to the outer membrane, which leads to polyubiquitination of damaged mitochondria to be cleared by lysosomal degradation (11). Mitophagy is one of the key adaptive responses to hypoxia where cells attempt to reduce their mitochondrial mass to limit ROS production as well as to augment efficient oxygen usage. BNIP3 and NIX are adaptor molecules that promote hypoxia-induced mitophagy (12). Both BNIP3 and NIX are directly activated by the hypoxia-inducible factors (HIFs) (12). Increasing evidence from various studies postulate that dysregulation of mitophagy is an etiological factor of cancer progression (13).

Mitochondrial membrane potential is impaired by ROS; irradiation or chemotherapeutic agents trigger the initiation of mitophagy, which involves PINK1 stabilization and Parkin recruitment (14, 15). Solid tumors of breast, ovarian, colon, and lung cancers were shown to harbor deletion or loss-of-function mutations in the PARK2/Parkin gene (16–18). As a selective type of autophagy, the formation of mitochondrial autophagosomes in mitophagy is also subject to the regulatory mechanisms of autophagy. Autophagy is a critical process in the early metastatic phase of breast cancer, and interestingly, proliferative breast cancer cells are resistant to autophagy inhibition (19). Autophagy inhibition results in the accumulation of defective mitochondria (20, 21).

Mitochondrial dysfunction modulates autophagy and more specifically the mitophagy response in cancer cells, given its role as a major site of ROS generation. Thus, understanding the relationship between autophagy and mitophagy will be essential. This study aims to assess the expression of protein markers of autophagy, mitophagy, oxidative stress, and apoptosis in invasive breast carcinoma tissues using immunohistochemistry.

## Materials and Methods

### Sample Collection

This study is registered to the National Medical Research Registry and ethical approval was obtained from the Malaysia Research and Ethics Committee (MREC), Ministry of Health, Malaysia, to preserve the anonymity and confidentiality of the patient (NMRR-18-2037-43079). Formalin-fixed, paraffin-embedded (FFPE) tissue samples from 100 breast cancer patients diagnosed in Hospital Kuala Lumpur, Hospital Putrajaya, National Cancer Institute, and National Cancer Institute were collected. The exclusion criteria included preoperative chemoradiotherapy. We have also excluded samples with missing data (*n* = 24); thus, the final number of samples included in this study is 76. After excluding patients according to these criteria, paraffin-embedded tissue blocks from both normal and tumor tissues were obtained from the Department of Pathology of the respective hospitals. All slides were reviewed retrospectively by breast pathologists. Clinicopathological parameters were assessed, which included patient age at initial diagnosis, sex, breastfeeding status, marital status, number of children, tumor location, histological form of tumor, tumor stage, and molecular subtype, and were obtained from clinical and pathological records. All patients were informed about the aim of the study, and a signed consent form that is approved by the ethical board was obtained from each recruited patient.

### Immunohistochemistry

#### Tissue Microarray

Tissue microarray (TMA) construction was performed at the Department of Pathology, UKM. Representative areas with tumor cells and non-tumorous areas from the FFPE tissue blocks were carefully selected based on pathology assessment of the H&E-stained slides and were used for TMA construction. The TMAs were assembled using a tissue arraying instrument (Alphelys Minicore 3 Tissue Arrayer). The instrument was used to create holes in a recipient paraffin block with defined array coordinates. Two representative areas of 1.0 mm in diameter tissue core were taken from each block. These tissue cores were arrayed into recipient paraffin blocks of 28 × 22 mm, with 2.0-mm spacing between the cores, creating a maximum of 8 × 8 dots in the different blocks consisting of 64 cores in a single block. The block was then heated for 5 min at 60°C and 3-µm sections were cut using Microm microtome (HM 340E, Thermo Scientific, USA) and mounted onto adhesive-coated slides. One section from each block was stained with H&E to ascertain the presence of tumor in the cores.

#### Immunohistochemical Staining

Immunohistochemistry studies on the oxidative stress [manganese superoxide dismutase (MnSOD)], autophagy (Beclin-1, LC3), mitophagy (BNIP3, Parkin), and apoptosis (cleaved caspase-3) markers were performed. FFPE tissue sections (3 μm) of matched control and tumor TMAs were dewaxed at 60°C and rehydrated with xylene and ethanol. Recovery of antigen was performed using sodium citrate buffer (pH 6.0). Immunohistochemistry (IHC) was performed according to the manufacturer’s guideline (VectaStain ABC kit, Vector Laboratories, USA). Briefly, endogenous peroxidase activity was blocked using 0.3% hydrogen peroxidase for 10 min followed by blocking serum for 30 min. The tissues were incubated overnight in primary antibodies at 4°C. Phosphate buffer saline (PBS) and normal tissues of kidney, liver, and spleen were used as negative and positive controls, respectively. The following antibodies were used for IHC: anti-MnSOD (1:200; Santa Cruz Biotechnology, UK), anti-Caspase-3 (1:300; Cell Signaling, UK), anti-Beclin-1 (1:50; Santa Cruz Biotechnology, UK), anti-LC-3 (1:800; Abcam, Cambridge, UK), anti-BNIP-3 (1:100; Abcam, Cambridge, UK), and anti-Parkin (1:100; Abcam, Cambridge, UK). Following the overnight incubation, the tissues were incubated with biotinylated secondary antibody for 30 min at room temperature and finally with peroxidase substrate to induce the peroxidase-catalyzed reaction. The slides were counterstained with hematoxylin and mounted in aqueous DPX.

#### Scoring

All slides were manually scored and five random sections of each TMA blot were chosen for the intensity score. All IHC was scored independently by at least two researchers blinded to patient clinicopathological and outcome data. When disagreement on staining interpretation occurred, the relevant slides were re‐reviewed by pathologists to reach a consensus opinion. A cutoff value of 1% or more positively stained nuclei and cytoplasm was used to define antibody expression. H-scores were calculated as H-score = ∑ (1 + *i*) pi, where *i* is the intensity score, and pi is the percentage of positively stained cells (22).

Briefly, the score is assigned as follows: 0 for no staining, 1 for weak cytoplasmic, 2 for moderate cytoplasmic, 3 for weak nuclear and cytoplasmic, 4 for strong cytoplasmic, 5 for strong nuclear, 6 for moderate cytoplasmic and nuclear, and 7 for strong cytoplasmic and nuclear.

### Statistical Analysis

Data were analyzed using Statistical Product and Service Solutions (SPSS) version 25.0. Wilcoxon signed-rank test was used for continuous and categorical variables of staining while Fisher’s exact test was used for demographic analysis. Associations between variables were calculated using the phi contingency coefficient. All *p*-values <0.05 were considered as statistically significant unless stated otherwise.

## Results

### Demographic Data

**Figure 1:**
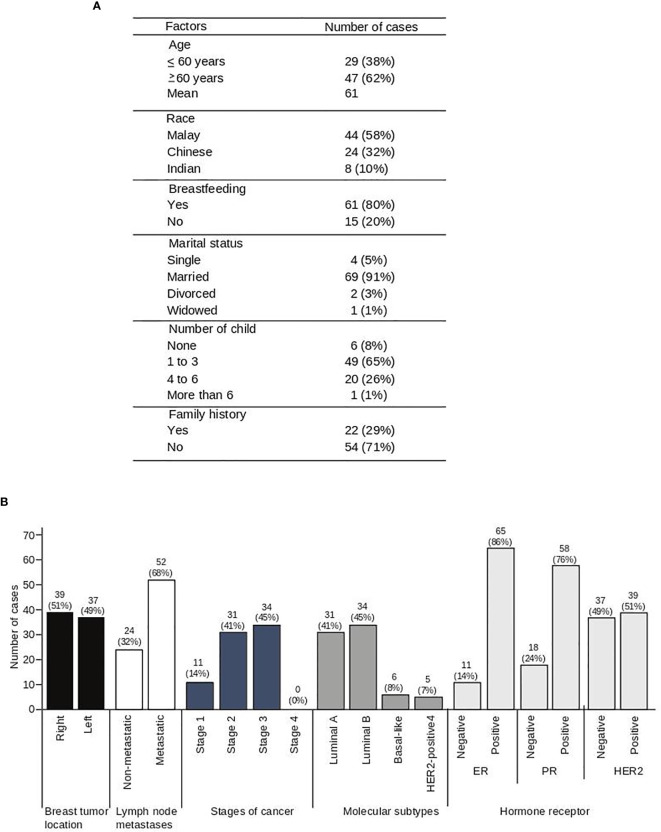
Socio-demographic data of 76 breast cancer patients. Demographic data **(A)** and clinical characteristics **(B)** of breast cancer patients.

A total of 76 samples were collected in this study, and the demographic data of the breast cancer patients enrolled are in **Figure 1**. The mean age was 61 ± 12 years. From the 76 patients, 4 patients were single and 72 patients were married once, while 71% of the patients had no family history of breast cancer (**Figure 1A**). Tumor metastases were noted in 68% of patients and tumor staging revealed that more than 86% of the cases are classified as Stage 2 and 3 breast cancers (**Figure 1B**). Of note, 41% and 45% of the patients were diagnosed with luminal A and luminal B breast cancer, respectively (**Figure 1B**). The hormone receptor expression in these patients revealed that 86% of the patients were positive for ER, 76% for PR, and 51% for HER2 (**Figure 1B**).

### Overexpression of Markers in Breast Cancer

**Figure 2:**
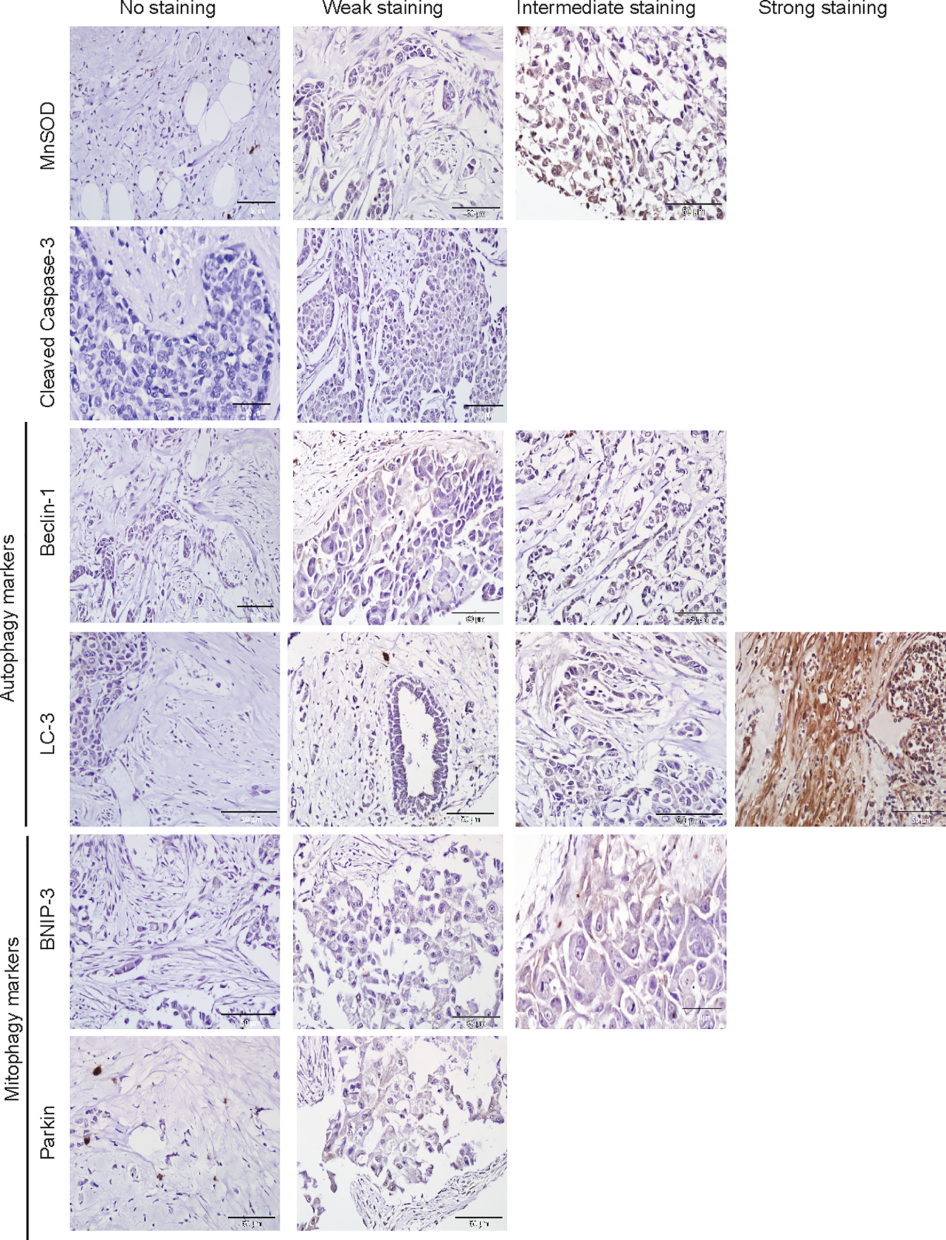
Immunohistochemical staining of MnSOD, cleaved caspase-3, Beclin-1, LC-3, BNIP-3, and Parkin in adjacent normal tissues. Representative images of MnSOD, cleaved caspase-3, Beclin-1, LC-3, BNIP-3, and Parkin staining (scored no, weak, intermediate, and strong staining) of adjacent matched control tissues obtained from breast cancer patient samples (*n* = 76).

**Figure 3:**
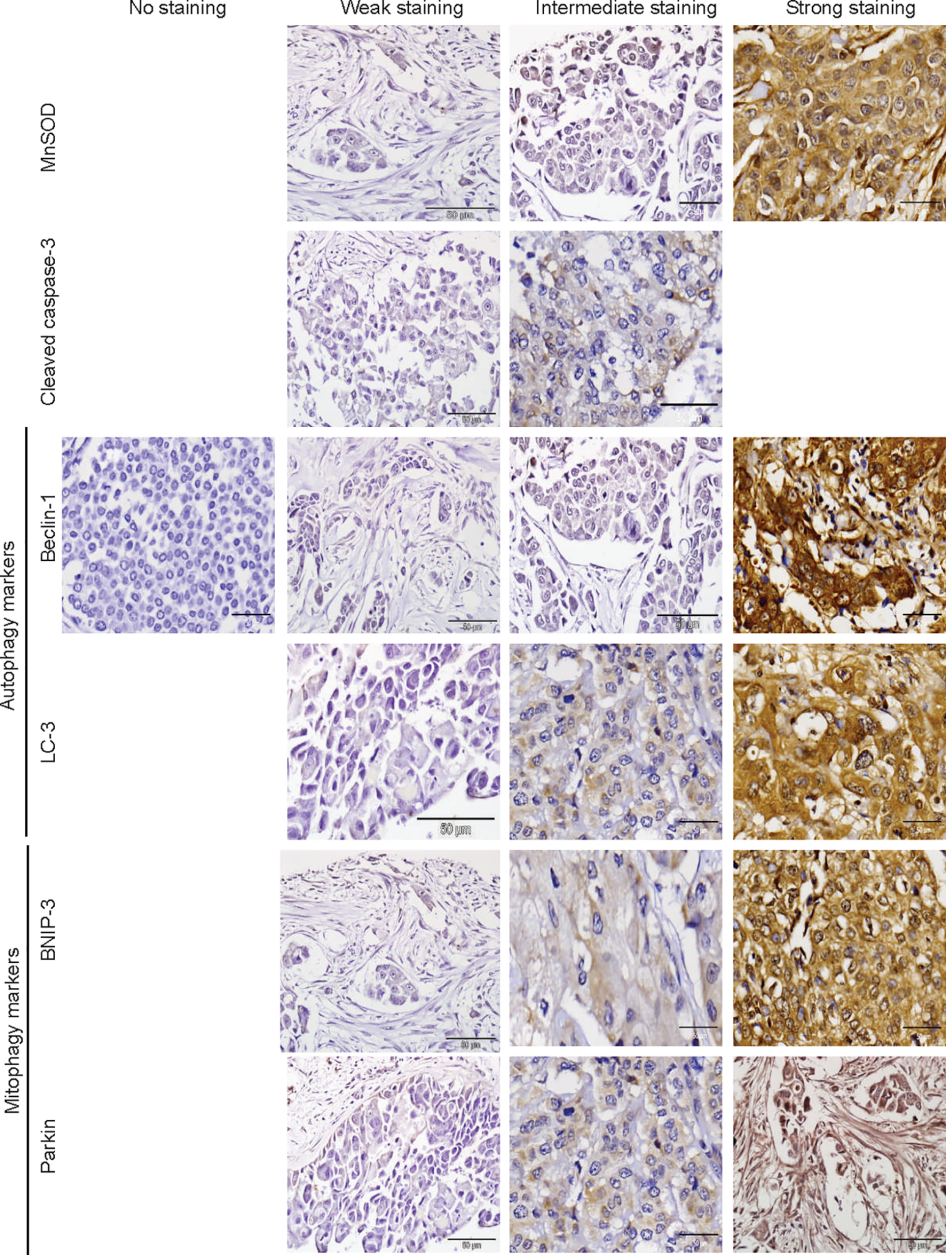
Immunohistochemical staining of MnSOD, cleaved caspase-3, Beclin-1, LC-3, BNIP-3, and Parkin in breast tumor tissues. Representative images of MnSOD, cleaved caspase-3, Beclin-1, LC-3, BNIP-3, and Parkin staining (scored no, weak, intermediate, and strong staining) of tumor tissues obtained from breast cancer patient samples (*n* = 76).

**Figure 4:**
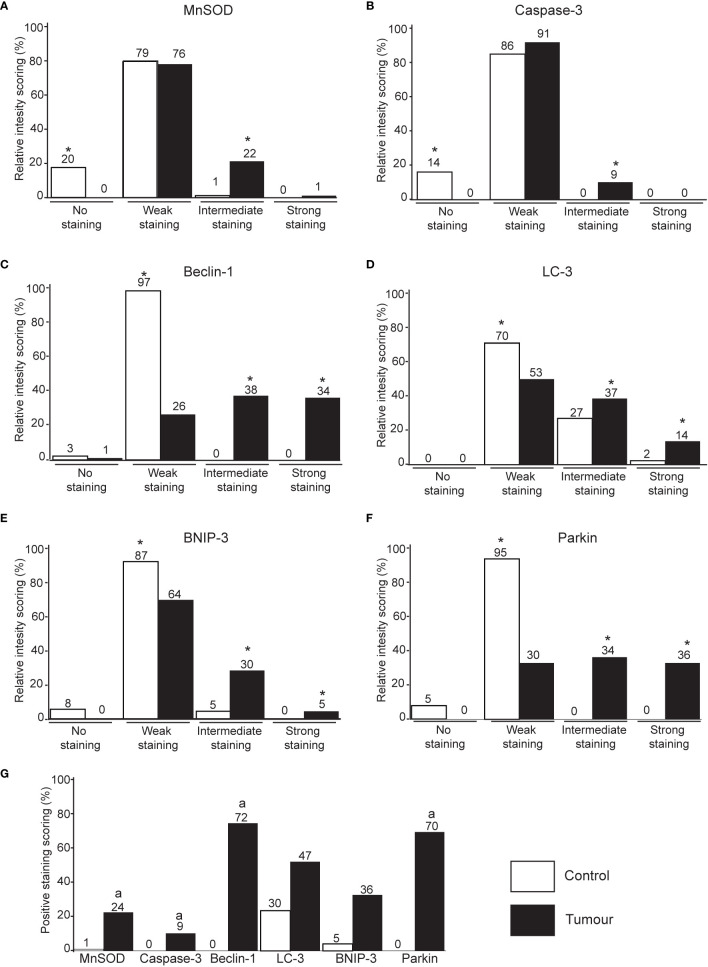
Immunohistochemical staining intensity score of MnSOD, cleaved caspase-3, Beclin-1, LC-3, BNIP-3, and Parkin in breast tumor tissues. Immunohistochemistry intensity score of MnSOD **(A)**, cleaved caspase-3 **(B)**, Beclin-1 **(C)**, LC-3 **(D)**, BNIP-3 **(E)**, and Parkin **(F)** in breast tissue. *p < 0.05 between all group pairs of samples. **(G)** The immunohistochemistry scoring intensity was further classified into tissues with positive staining (intermediate and strong staining) for all protein markers. ^a^p < 0.05 significantly lower staining between the protein markers.

The protein markers for oxidative stress, apoptosis, autophagy, and mitophagy were noted to be predominantly localized in the cytoplasm (**Figures 2** and **3**). In adjacent matched control tissues, MnSOD and cleaved caspase-3 were highly scored as no staining (**Figures 4A, B**). Beclin-1, LC-3, BNIP-3, and Parkin were highly scored as weak staining compared to tumor tissues (*p* < 0.05) (**Figures 4C–F**).

Tumor tissues stained with the oxidative stress (MnSOD) and apoptosis (cleaved caspase-3) markers are significantly scored as intermediate staining (**Figures 4A, B**). Intermediate and strong staining scores of Beclin-1, LC-3, BNIP-3, and Parkin were significantly higher in tumor tissues compared to the adjacent matched control (**Figures 4C–F**).

The scoring intensity was further classified into tissues with negative staining (scored as no staining and weak staining) and positive staining (intermediate and strong staining). Quantitative comparison showed that LC-3 was positively stained in adjacent matched normal tissues compared to other markers (*p* < 0.05). The positive scores of Beclin-1 and Parkin were significantly high in tumor tissues compared to other markers (**Figure 4G**).

BNIP-3 and Beclin-1 as well as LC-3 and cleaved caspase-3 immunostaining are positively associated (**Table 1**). Interestingly, MnSOD, cleaved caspase-3, Beclin-1, LC-3, BNIP-3, and Parkin are inversely associated (**Table 1**).

**Table 1:** Association between various protein markers.

	MnSOD	Caspase-3	Beclin-1	LC-3	BNIP-3	Parkin
MnSOD	1#	0.175 (0.138)	0.003 (0.603)	0.153 (0.143)	−0.026 (0.529)	0.030 (0.521)
Caspase-3		1#	0.007 (1.000)	**0.289 (0.012)**	0.140 (0.410)	0.205 (0.094)
Beclin-1			1#	0.174 (0.199)	**0.397 (0.000)**	0.105 (0.258)
LC-3				1#	0.067 (0.366)	-0.178 (0.096)
BNIP-3					1#	0.010# (1.000)
Parkin						1#

# indicates phi contingency coefficient value.

p-values are showed in parenthesis.

p < 0.05 were highlighted in bold.

### Correlation Between Markers Expression and Clinicopathologic Factors of Breast Cancer

LC-3-positive immunostaining was positively associated with a younger breast cancer patient (less than 60 years old) (*p* < 0.05). Parkin, which is positively immunostained, was positively associated with breast-feeding patients compared to non-breast-feeding patients (**Table 2**). A positive association was also noted between MnSOD immune-negative staining and marital status of the breast cancer patients. No significant association was identified between protein markers expression and race, number of children, location of tumor, lymph node involvement, stages of cancer, molecular subtype classification, and hormone receptor expression.

**Table 2:** Correlation analysis of MnSOD, Caspase-3, Beclin-1, LC-3, BNIP-3, and Parkin expression and clinicopathological factors.

Factors	BNIP-3		Parkin		LC-3		Beclin-1		MnSOD		Caspase-3	
	+	-	+	-	+	-	+	-	+	-	+	-
*Age*												
*p*-value	0.88		0.91		**0.01**		0.29		0.24		0.79	
<60 years	10	19	20	9	19	10	19	10	9	20	3	26
≥60 years	17	30	33	14	17	30	36	11	9	38	4	43
*Race*												
*p*-value	0.60		0.16		0.98		0.91		0.97		0.70	
Malay	14	30	27	17	21	23	31	13	10	34	3	41
Chinese	9	15	19	5	11	13	18	6	6	18	3	21
Indian	4	4	7	1	4	4	6	2	2	6	1	7
*Breastfeeding*												
* p*-value	0.69		**0.03**		0.61		0.46		0.76		0.70	
Yes	21	40	39	22	28	33	43	18	14	47	6	55
No	6	9	14	1	8	7	12	3	4	11	1	14
*Marital status*												
* p*-value	0.16		0.34		0.82		0.33		**0.03**		0.85	
Single	3	1	4	0	2	2	3	1	2	2	0	4
Married	22	47	46	23	33	36	50	19	14	55	7	62
Divorced	1	1	2	0	1	1	2	0	2	0	0	2
Widowed	1	0	1	0	0	1	0	1	0	1	0	1
*Number of children*												
* p*-value	0.34		0.17		0.54		0.85		0.18		0.24	
None	4	2	6	0	2	4	5	1	3	3	0	6
1 to 3	17	32	33	16	22	27	35	14	13	36	7	42
4 to 6	6	14	14	6	11	9	14	6	2	18	0	20
More than 6	0	1	0	1	1	0	1	0	0	1	0	1
*Location of tumor*												
* p*-value	0.17		0.92		0.11		0.69		0.23		0.64	
Right	11	28	27	12	15	24	29	10	7	32	3	36
Left	16	21	26	11	21	16	26	11	11	26	4	33
*Lymph node involvement*												
* p*-value	0.20		0.50		0.50		0.84		0.33		0.50	
Metastatic	16	36	35	17	26	26	38	14	14	38	4	48
Non malignancy	11	13	18	6	10	14	17	7	4	20	3	21
*Stage of cancer*												
* p*-value	0.43		0.17		0.35		0.39		0.85		0.99	
Stage 1	2	9	9	2	3	8	7	4	3	8	1	10
Stage 2	12	19	24	7	15	15	25	6	8	23	3	28
Stage 3	13	21	20	14	17	17	23	11	7	27	3	31
Stage 4	0	0	0	0	0	0	0	0	0	0	0	0
*Molecular subtype*												
* p*-value	0.15		0.26		0.94		0.55		0.61		0.71	
Luminal A	13	18	23	8	14	17	22	9	8	23	3	28
Luminal B	8	26	22	12	16	18	24	10	6	28	4	30
Basal-like	4	2	3	3	3	3	4	2	2	4	0	6
HER2 positive	2	3	5	0	3	2	5	0	2	3	0	5
*Hormone receptor*												
*ER*												
* p*-value	0.18		0.56		0.75		0.72		0.23		0.58	
Negative	6	5	8	3	6	5	9	2	4	7	0	11
Positive	21	44	45	20	30	35	46	19	14	51	7	58
*PR*												
* p*-value	0.17		0.77		0.80		0.56		0.57		0.47	
Negative	9	9	12	6	8	10	12	6	4	14	1	17
Positive	18	40	41	17	28	30	43	15	14	44	6	52
*HER2*												
* p*-value	0.09		0.56		0.82		0.80		0.59		0.53	
Negative	17	20	26	11	17	20	26	11	10	27	3	34
Positive	10	29	27	12	19	20	29	10	8	31	4	35

p < 0.05 were highlighted in bold.

## Discussion

One of the hallmarks of cancer that gained significant attention as a therapeutic target in the past decade is the altered metabolism observed in tumor pathogenesis that allows cancer survival (23). Warburg’s seminal discovery on the role of mitochondria and aerobic glycolysis serves as the fundamental stepping stone in the involvement of mitochondria in tumorigenesis (24). Mitochondria play a central role in metabolic reactions and drive this rewiring *via* various pathways that are influenced by several factors such as metabolic state, tumor heterogeneity, types of tissues involved, and tumor stage (25, 26). Tumor metabolism involves energy generation to fuel biochemical reactions, which provide building blocks to support cell growth as well as to sustain biochemical homeostasis that includes maintenance of redox potential (27).

Extensive clinical research reported that alterations in metabolic profiles were observed mainly in pathways involving Krebs cycle, glycolysis, amino acids, nucleotide, and/or lipid metabolism of breast cancer and normal samples (28–30). Several preclinical data demonstrated the involvement of ROS, mitophagy, and autophagy in breast cancer progression. Impaired mitophagy and increased levels of mitochondrial superoxide radicals were shown to affect *in vitro* breast cancer cell proliferation (31, 32). In a mouse model of mammary tumorigenesis, inactivation of BNIP3 resulting in defective mitophagy was shown to elevate ROS production and normoxic HIF-1α stabilization, which accelerated tumor progression to metastasis (33). Interestingly, limited data on mitophagy and ROS levels in breast cancer patient are reported. This study demonstrates the levels of ROS, mitophagy, and autophagy in a breast cancer patient without any prior history of chemo- and radiotherapy treatment.

MnSOD level was significantly higher in tumor tissues compared to matched control (**Figure 4**), which is in agreement with other studies reporting on breast cancer patients (34). Several studies have reported that overexpression of MnSOD positively regulates cancer progression from a localized to an invasive phenotype (35, 36) and tumor cell adhesion (37, 38) in a panel of breast cancer cells. *In vivo* and *in vitro* studies demonstrated Nrf2-driven MnSOD upregulation postulated from degradation of Caveolin-1 resulted in enhanced cell survival, metastasis, and drug resistance in circulating breast cancer cells (39, 40).

The caspase cascades play pertinent roles in apoptotic induction and is associated with tumorigenesis. Studies have reported that high caspase-3 expression is associated with adverse survival in breast cancer patients (41, 42) and other cancers that include gastric, ovarian, cervical, colorectal, and lung cancer (43, 44). Interestingly, it was also suggested that loss of caspase-3 expression is an important cell survival mechanism in breast cancer patients (45). The role of caspases in cancer development remain a double-edged sword. This study reports that cleaved caspase-3 staining is significantly higher in tumor tissues compared to adjacent matched control tissues (**Figure 4B**). Several studies have implicated the association of caspase-3 expression and clinical outcomes in various cancers (46, 47). However, no significant association was observed between cleaved caspase-3 expression and clinicopathological data.

Beclin-1 was reported as a haploinsufficient tumor suppressor gene that participates in the early stage of autophagosome formation (48) in contrast to LC-3, which is a marker for final autophagasome formation. High LC-3 protein levels were associated with tumorigenesis in TNBC patients (49, 50). The association of high LC3 expression with poor outcomes was reported in other cancers, which include colorectal (51), gastric (52), malignant melanoma (53), and esophageal (54). Increased Beclin-1 expression was noted in tamoxifen-resistant breast cancer cell line (55). There are several conflicting studies that report the correlation between Beclin-1 expression and breast cancer prognosis. Reduced Beclin-1 expression was associated with poor overall survival and distant metastasis-free survival (56) and high expression was associated with good prognosis (57). However, there are studies that found no correlation between Beclin-1 expression and breast cancer patient prognosis (58, 59).

The observation of significantly high Beclin-1 in tumor compared to adjacent matched tissues in contrast to LC-3 expression that showed no significant difference might suggest that the tumorigenesis in these patients is associated with a defective autophagic process (60). Autophagy plays a dual role in cancer whereby at an early stage of cancer formation, it plays a quality control role by removing defective proteins. However, once the cancer has progressed to late stage, it mediates tumor promotion and causes resistance to chemotherapeutic agents (61). The staging of breast cancer patients in this study is categorized according to TNM staging ranging from stage 1 to 3, which suggests they are not advanced tumors (**Figure 1**). Hence, it is postulated that the high basal autophagy noted in these tumor samples indicated by increased Beclin-1 expression promotes tumor progression as well as treatment resistance. Of note, this study is also in agreement with the association between cleaved caspase-3 and LC-3 (**Table 1**), which suggests that dysregulated autophagy promotes caspase-dependent apoptosis.

Parkin expression is inversely correlated with poorly differentiated grades of breast cancer (62, 63). Parkin is well known as a tumor suppressor protein that inhibits tumor cell growth. The upregulation of Parkin observed in this study suggests the negative regulation of Parkin on cancer cell metastasis (64, 65). The local spread of cancer indicated by staging in this study supports the role of Parkin impairing the migration of cancer cells (66). Increase in BNIP-3 expression promotes autophagosome accumulation with lysosome consumption (67, 68). BNIP-3 deletion was most commonly found in TNBC and is associated with perturbed mitophagy that leads to increased invasiveness and metastasis (33). Reduced BNIP-3 expression observed in invasive breast cancer is correlated with poor prognosis characterized by high proliferation and positive lymph node status (69, 70). However, although upregulation of BNIP-3 is associated with good survival outcome in invasive breast carcinoma, it is also linked to an increased risk of recurrence and shorter disease-free survival in DCIS (71).

The findings in this study measure the basal autophagic and mitophagic status at the time of diagnosis and did not evaluate the expression of these markers after therapeutic intervention. To date, this is the first study that measured oxidative stress, mitophagy, and autophagy markers in the same breast cancer tissues and adjacent matched control. The breast cancer samples in this study are categorized to an early stage of cancer development that involves local spread. The findings in this study are in agreement with the initial role of autophagy and mitophagy in cancer that were involved in the programmed removal of defective proteins and mitochondria. However, owing to the small sample size, the expression of autophagy and mitophagy in breast cancer tissues warrants further investigation.

## Data Availability Statement

The raw data supporting the conclusions of this article will be made available by the authors, without undue reservation.

## Ethics Statement

This study is registered to the National Medical Research Registry and ethical approval was obtained from the Malaysia Research and Ethics Committee (MREC), Ministry of Health Malaysia to preserve the anonymity, confidentiality of the patient (NMRR-18-2037-43079). The patients/participants provided their written informed consent to participate in this study.

## Author Contributions

MFM, MAA, and SM participated in the design of the research; MAA and SM guided the group of researchers. MFM, NHNY and NRG were involved in sample collection whilst MFM, NMSTL, and SHMP contributed in TMA construction. MFM carried out the experiments and analysed the data with the guidance of SMS under the supervision of MAA and SM. MFM and SM wrote the paper; SF and MAA critically revised the manuscript. All authors contributed to the article and approved the submitted version.

## Funding

The work was supported by the Ministry of Education Malaysia (Programme Grant: Fundamental Research Grant Scheme FRGS/1/2015/SKK02/UPM/02/4) and Universiti Putra Malaysia (Programme Grant: Putra Grant GP-IPS/2017/9576800).

## Conflict of Interest

The authors declare that the research was conducted in the absence of any commercial or financial relationships that could be construed as a potential conflict of interest.

## Publisher’s Note

All claims expressed in this article are solely those of the authors and do not necessarily represent those of their affiliated organizations, or those of the publisher, the editors and the reviewers. Any product that may be evaluated in this article, or claim that may be made by its manufacturer, is not guaranteed or endorsed by the publisher.

## Abbreviations

ERα, Estrogen receptor α; FFPE, Formalin-fixed, paraffin-embedded; HER2, Human epidermal growth factor receptor 2; HIF, Hypoxia-inducible factor; IHC, Immunohistochemistry; MnSOD, Manganese superoxide dismutase; MREC, Malaysia Research and Ethics Committee; PBS, Phosphate buffer saline; PR, Progesterone receptor; ROS, Reactive oxygen species; TMA, Tissue microarray; TNBC, Triple-negative breast cancer; TNM, Tumor node metastasis.
